# Deciphering the nutritive and antioxidant properties of Malay cherry (*Lepisanthes alata*) fruit dominated by ripening effects

**DOI:** 10.1039/c9ra05312c

**Published:** 2019-11-21

**Authors:** Yan Zhang, Shufei Chen, Junwei Huo, Dejian Huang

**Affiliations:** College of Horticulture and Landscape Architecture, Northeast Agricultural University Heilongjiang 150030 China huojunwei@neau.edu.cn; Department of Food Science and Technology, National University of Singapore 3 Science Drive 3 Singapore 117543 Singapore chmhdj@nus.edu.sg +65 6775 7895 +65 6516 8821; National University of Singapore (Suzhou) Research Institute, Suzhou Industrial Park Jiangsu 215123 China

## Abstract

In this study, Malay cherry fruit were explored for the changes in their nutritive and phenolic compositions upon ripening (unripe and ripe stages). Nutritive compositions (sugars, proteins, and fats) of the fruit increased, whilst organic acids of the fruit decreased in ripe fruit. Twenty-eight non-anthocyanin phenolics of the fruit were identified by the high-performance liquid chromatography-high resolution-time of flight mass spectrometry (HPLC-HR-TOF/MS^2^). Among them, quercetin-3-*O*-rutinoside and quercetin-3-*O*-glucoside are dominant species in the unripe fruit, and four more phenolics are shown in the ripe fruit. Additionally, seventeen anthocyanins were solely identified in the ripe fruit. This could be the signature phenolic profile of Malay cherry fruit. The total phenolics and total proanthocyanidins of the fruit significantly decreased upon ripening. Consistently, antioxidant capacities of the fruit also decreased upon ripening. Our results suggest unripe fruit are good sources of phenolic antioxidants that are worthwhile for utilisation as functional food sources.

## Abstract 2

In this study, Malay cherry fruit were explored for the changes in their nutritive and phenolic compositions upon ripening (unripe and ripe stages).

## Graphical abstract

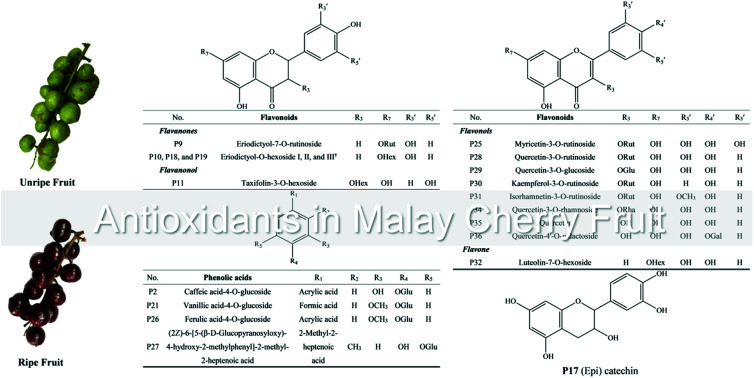

## 1. Introduction

From ancient to modern times, ripe fruit are more acceptable to human beings than unripe ones. This could be simply because the ripe fruit always give a more enjoyable sensory experience. Knowledge of nutrients (sugars, organic acids, proteins, and fats) and phenolics in ripe and unripe fruit is likely to be ignored despite their large differences. McCune *et al.* revealed the fact that nutrients and phenolics of fruit are dominated by their ripening stages.^1^ A growing body of literature has, in fact, demonstrated that ripe sweet cherry,^2^ red raspberry,^3^ and blueberry^4^ possess higher total phenolic contents (TPC) than unripe ones; however, the contrary was observed in the TPC of black raspberry,^3^ strawberry,^3^ and blackberry.^5^. In other words, the changes in the TPC of ripe fruit are unpredictable. Therefore, an individual study is necessary to understand the nutritive and phenolic changes in a specific fruit upon ripening.

The ripening of fruit includes three distinct stages: unripe, veraison, and ripe stages. As the nutritive and phenolic compositions of fruit vary largely at the intermediate stage, it is hard to be tracked. Therefore, the two steady stages (unripe and ripe) of fruit are expected to be further studied.^6^

Fruit are great sources of phenolic antioxidants that are believed to have wide spectrum of health benefits. This is especially so for berries and cherries. The berries are rich in phenolic antioxidants of diverse chemical structures and health promotion effects.^7^ We have witnessed the market penetration of acai berry from South America to become mainstream health foods because of their high antioxidant contents.^8^ Similarly, tart cherries are also known to contain high phenolic antioxidants with anti-inflammatory properties that can help reducing pain and accelerating strength recovery after exercise.^9^ It is well known that the phenolics of cherries support the potential preventive health benefits of cherry intake in relation to cancer, cardiovascular disease, inflammatory disease, diabetes, and Alzheimer's disease.^1^ There are many more small fruit, cultivated or wild, that receive little or no attention in scientific community and their potential values await to be uncovered as functional food ingredients. This is especially so in tropical region where there are plenty of exotic fruit. One such example is *Lepisanthes alata*, commonly known as Malay cherry. *L. alata* is a tropical species of Sapindaceae native to Southeast Asia. The fruit of Malay cherry are sub-globose shaped (2–4 cm) with the colour from green to dark reddish purple as fruit ripen. The pulp-with-peel portions of the ripe fruit are eaten fresh with fairly sweet taste.^10^ While the Malay cherry has long been popular in Singapore as a landscape tree, it attracted international interest resulting from our first report.^11^ We are intrigued by this tree because we found that the fruit and leaf proanthocyanidins showed potent inhibitory activity against starch hydrolases.^11^ According to our preliminary study, Malay cherry fruit are rich in not only proanthocyanidins, but also in other phenolic compounds (polyphenols and simple phenolics). Among these phenolic compounds, a continuously vast interest has been focused on anthocyanins and polyphenols due to their relatively high antioxidant capacities and corresponding health benefits.^2,12^ However, in terms of Malay cherry fruit, there is virtually no information on the profile of phenolic compounds, not to mention phenolic changes upon ripening. To our best knowledge, this is the first study to evaluate the effect of ripening on the nutritive and antioxidant properties of Malay cherry fruit.

From a food product design standpoint, nutritive and phenolic compositions, which largely determine the functionality of foods, can be modulated by ripening. Therefore, it is a prerequisite to be clear on the nutritive and phenolic compositions of the unripe and ripe fruit of Malay cherry. As well, additional understandings of the changes in total phenolics, total anthocyanins, total proanthocyanidins, and antioxidant capacities of these fruit were of great importance. Therefore, the aim of this study was to investigate the above descriptions. More specifically, unripe and ripe fruit were subjected to assess their antioxidant capacities and consequently identify the major phenolic compounds within the fruit.

## 2. Materials and methods

### 2.1 Materials

The Folin–Ciocalteu reagent, gallic acid, potassium chloride, sodium acetate, 4-dimethylaminocinnamaldehyde (DMAC), procyanidin A2, Trolox, 2,2′-azobis (2-methylpropionamidine) dihydrochloride (AAPH), fluorescein, potassium dihydrogen phosphate, dibasic potassium phosphate, total dietary fibre assay kit (TDF-100A), sodium phosphate, catalyst mixture (47.7% anhydrous sodium sulphate, 47.7% potassium sulphate, 2.8% titanium dioxide, and 1.8% copper sulphate), methyl red, boric acid, 3,5-dinitrosalicylic acid (DNSA), maltose, glucose, fructose, sucrose, malic acid, succinic acid, citric acid, ammonium acetate, and sodium acetate were purchased from Sigma-Aldrich (St. Louis, MO). Sulphuric acid (98%), hydrochloric acid (37%), phosphoric acid (85%), and analytical grade solvents were purchased from Merck (Darmstadt, Germany). Sodium carbonate was purchased from GCE Chemicals (Malmö, Sweden). Acetic acid (glacial) was purchased from RCI Labscan (Bangkok, Thailand). HPLC grade solvents were purchased from VWR International GmbH (Darmstadt, Germany).

The fresh Malay cherry fruit were sampled from 20 multiple trees at unripe (green) and ripe (dark reddish purple) stages on 9 and 23 October in Singapore, respectively. The sampling area is a 1 km radius around 1.449 N 103.820 E in Sembawang district of Singapore. Over 100 fruit were sampled at each stage for further study. After removal of the seeds of fruit, only the pulp-with-peel portions of fruit were used in this study. The phytonutrient contents in the seeds will be studied separately.

### 2.2 Physicochemical analysis

#### 2.2.1 Nutritive compositions

The moisture contents of fresh unripe and ripe fruit were determined according to AOAC Official Method 930.04. The fresh fruit were lyophilised using an advantage benchtop tray lyophiliser (the VirTis Company, Inc., Gardiner, NY) and ground into fine powders with a mini blender (DM-6, YOUQI, Changhua, Taiwan). The fine powders obtained were stored at −20 °C until further determinations. The ash, protein, fat, insoluble dietary fibre, and soluble dietary fibre contents of fruit powders were determined according to AOAC Official Method 930.05, 977.02, 954.02, and 985.29.^13^ The total dietary fibre of fruit powders was calculated as combined values of the insoluble and soluble dietary fibres.

The sugar contents and compositions of unripe and ripe fruit were evaluated as follows. The defatted and lyophilised fruit powder (1.0 g) was ultrasonicated with aqueous ethanol (10.0 mL, 80%, v/v) for 30 min. The slurry was centrifuged at 12 074 g for 10 min at 4 °C to get the supernatant followed by evaporation at 40 °C. The concentrated sugar extracts were diluted with deionised water in the ratio of 1 : 1 to get the ethanol–free sugar extracts (4 mL) for the following measurement. The total soluble solids (TSS) of the sugar extracts were measured using a digital RX-5000α refractometer (ATAGO, Tokyo, Japan) and calibrated using deionised water at 20 °C. The TSS was expressed as percent sucrose. The reducing sugar contents of the sugar extracts were measured using the DNSA assay and expressed as milligram maltose equivalent per gram of dry weight of fruit, according to our previous study.^14^ In brief, 100 μL of DNSA reagent was mixed with 100 μL of sugar extracts or maltose standard solutions (0 to 1.5 g L^−1^) in a 96-well microplate and boiled for 5 min. 100 μL of the cooling mixture was transferred to another 96-well microplate for measuring the absorbance at 540 nm with a Synergy HT microplate reader (Biotek Instruments Inc., Winooski, VT). The sugar compositions of the sugar extracts were analysed by a ultrafast liquid chromatograph Prominence system coupled with a LTII evaporative light scattering detector (Shimadzu, Kyoto, Japan), according to a literature method.^15^ Serial diluted sugar extracts (10 μL) or standards (fructose, glucose, and sucrose ranging from 0.1 to 10.0 g L^−1^) were filtered through a regenerated cellulose filter (0.45 μm) and injected into a Zorbax carbohydrate column (4.6 × 150 mm, 5 μm) with a guard column made by the same materials (Agilent, Palo Alto, CA). Mobile phase was acetonitrile (80%, v/v) at a flow rate of 1.4 mL min^−1^ for 23 min. The column oven temperature was set at 40 °C. Detector was set at 40 °C, gain 5, and pressure of 350 kPa. Sugars in the sample were identified by matching the retention times of standards and their concentrations were calculated by peak areas of the standard curves of the respective sugars. The standard curves were plotted with *R*^2^ greater than 0.99. The total sugar contents of fruit were calculated as combined values of fructose, glucose, and sucrose.

The organic acids of unripe and ripe fruit were quantified as follows. The fruit powder (1.0 g) was ultrasonicated with deionised water (10.0 mL) for 30 min. The slurry was centrifuged at 12 074 g for 10 min at 4 °C to get the supernatant. The supernatant was filtered and diluted with deionised water up to 25 mL in a volumetric flask. Organic acids of the solution were identified and quantified by ultrafast liquid chromatograph Prominence system coupled with a photodiode array detector (PDA, Shimadzu, Kyoto, Japan), according to a literature method.^15^ The solution (10 μL) was injected into a Supelcogel C-610H ion exchange column (7.8 mm × 300 mm, Supelco, Inc., Bellefonte, PA). The mobile phase was 0.10% H_2_SO_4_ at an isocratic flow rate of 0.40 mL min^−1^ for 50 min at 40 °C. Standards were prepared with serial concentrations of malic, succinic, and citric acids at 0.02–10.00 g L^−1^. The absorbance was monitored at 210 nm. The concentrations of respective standards were calculated from calibration curves. All the curves had good linearity fit (*R*^2^ > 0.99). A FE20 K pH meter (Mettler Toledo, Switzerland) was used for pH measurements of unripe and ripe fruit.

#### 2.2.2 Colour, dimension, and weight measurements

The colours of fresh fruit peel were measured using a CM-5 spectrophotometer (Konica Minolta, Tokyo, Japan) equipped with a D65 illuminant based on the CIE 1976 (L*, a*, b*) colour space. The specular component excluded (SCE) mode with a 3 mm Petri dish was used for all measurements. The dimension (length and diameter) and weight measurements of intact fresh unripe and ripe fruit were carried out.

### 2.3 Extraction and purification of phenolic compounds

The unripe and ripe fruit powders (20.0 g) were separately extracted with methanol (80%, v/v, 2 × 100 mL) for 2 h by shaking on a vortex shaker. Each slurry was centrifuged and then each supernatant was evaporated at 40 °C to obtain crude extracts for solid-phase extraction (SPE).

The phenolic compounds were purified by SPE according to a literature method.^16^ The C18 Sep-Pak cartridges (Waters, Wexford, Ireland) were preconditioned sequentially with ethyl acetate (10 mL), methanol (10 mL), and 0.01 M HCl (15 mL). The crude extract (1 mL, approximately 0.5 g L^−1^) was loaded on the C18 cartridge that was eluted with 0.01 M HCl (15 mL). The adsorbed non-anthocyanin phenolics were eluted with ethyl acetate (40 mL). The adsorbed anthocyanins were then eluted with acidic methanol (0.1% HCl in methanol, v/v) until the eluent turned colourless. All eluents were separately evaporated at 40 °C and filtered for characterisation and antioxidant capacity.

### 2.4 Characterisation of phenolic compounds using HPLC-PDA and HPLC-HR-TOF/MS^2^

Samples (20 μL) were injected into a 2695 high-performance liquid chromatography (HPLC) coupled with a 2669 PDA detector (Waters, Milford, MA) and a HPLC coupled with micrOTOF-Q II high resolution time of flight mass spectrometry (HR-TOF/MS^2^, Bruker, Billerica, MA) with a reversed-phase C18 Sunfire column (250 mm × 4.6 mm i.d., 5 μm, Waters, Wexford, Ireland).

For the analysis of non-anthocyanin phenolics, the mixture were eluted with the ternary mobile phases consisting of A (50 mM aqueous ammonia acetate, pH 3.6), B (20% A in acetonitrile, v/v, pH 3.6), and C (200 mM acetate acid, pH 2.6). Elution programme started with 14% B and 86% C, changing to 16.5% B and 83.5% C at 12.5 min, 25% B and 75% C at 17.5 min, 80% B and 20% C at 40 min, and washing with 100% A for another 20 min. The flow rate and temperature were set at 1.0 mL min^−1^ and 25 °C. Anthocyanins were eluted with the binary mobile phases consisting of A (acetonitrile) and B (acidic water). Mobile phase B was prepared with 10% acetic acid and 5% acetonitrile, by volume. Elution programme started with 100% B for 5 min, decreasing to 80% B at 20 min, 60% B at 25 min, ramping up to 100% B at 30 min, and holding for 5 min at a flow rate of 1.0 mL min^−1^ at 25 °C.

HR-TOF/MS^2^ analyses were performed using a TOF mass spectrometer *via* electrospray ionisation (ESI) interface and controlled by Compass Data Analysis software. Mass spectra were acquired in negative mode for non-anthocyanin phenolics and in positive mode for anthocyanins with the range of *m*/*z* 50–1500. MS calibration standard was performed using sodium acetate. The MS^2^ collision gas was nitrogen. The negative ion ESI parameters were capillary voltage 3500 V, dry gas temperature 200 °C, dry gas flow 7.0 L min^−1^, and nebuliser 3.0 bar. The positive ion ESI parameters were capillary voltage 4500 V, dry gas temperature 200 °C, dry gas flow 7.0 L min^−1^, and nebuliser 3.0 bar.

### 2.5 Quantification of total phenolic, anthocyanin, and proanthocyanidin contents

The unripe and ripe fruit powder (1.0 g) was separately extracted with methanol (80%, v/v, 2 × 5 mL) for 2 h and centrifuged to get the supernatant. The supernatant was filtered and diluted with 80% methanol up to 25 mL in a volumetric flask. The solution was used for further analysis. The total phenolic content (TPC) of fruit was measured using the Folin–Ciocalteu assay with slight modifications.^17^ The solutions with serious dilutions (20 μL) were mixed with deionised water (90 μL) and Folin–Ciocalteu reagent (10 μL) in a 96-well plate and incubated for 5 min at room temperature in the dark. The Na_2_CO_3_ solution (80 μL, 75 g L^−1^) was added to the mixture and incubated for another 2 h. The absorbance was captured at 765 nm using the microplate reader. A calibration curve of gallic acid was constructed yielding a linear correlation (*y* = 4.8595*x* + 0.0047) with a high *R*^2^ value of 0.99. The TPC of fruit was expressed as milligram gallic acid equivalent per gram of dry weight of fruit.

The total anthocyanin content (TAC) of fruit was separately measured according to the pH differential method with slight modifications of a reported method.^18^ Potassium chloride buffer (0.025 M, pH 1.0) and sodium acetate buffer (0.4 M, pH 4.5) were prepared for dilution. Two diluted extracts (20 μL) were mixed with corresponding buffer (180 μL) in a 96-well plate and the absorbance of each well was read at 510 nm and 700 nm using the microplate reader. Absorbance was calculated with the aid of eqn (1) below. The TAC of fruit was calculated using following eqn (2) and expressed as milligram cyanidin-3-*O*-glucoside equivalent per gram of dry weight of fruit.

1 *A* = (*A*_510 nm_ − *A*_700 nm_)_pH1.0_ − (*A*_510 nm_ − *A*_700 nm_)_pH4.5_

2 Cyanidin-3-*O*-glucoside equivalent (mg g^−1^ of dry weight of fruit) = (*A* × MW × DF × 1000)/(*ε* × 1)

where *A* is absorbance; MW is the molecular weight of cyanidin-3-*O*-glucoside (449.2); DF is dilution factor; and *ε* is molar absorptivity (26 900).

The total proanthocyanidin content (TPAC) of fruit was measured using the DMAC assay with slight modifications.^19^ The extracts were serial diluted with a mixture [80% of ethanol (91%): 20% of deionised water, v/v] and pipetted (70 μL) into a 96-well plate to mix with fresh DMAC solution (210 μL, 0.1% DMAC in acidified ethanol, w/v). The acidified ethanol was prepared by mixing 75% of ethanol (91%), 12.5% of deionised water, and 12.5% of HCl (36%), by volume. The absorbance was read for 30 min using the microplate reader at 640 nm at 25 °C. The maximum absorbance was calculated using a pre-determined procyanidin A2 calibration curve (*y* = 24.303*x* + 0.0151) with *R*^2^ value of 0.99, where *y* represents the absorbance value and *x* represents the procyanidin A2 concentration (g L^−1^). The results were expressed as milligram procyanidin A2 equivalent per gram of dry weight of fruit.

### 2.6 Antioxidant capacity analysis

Antioxidant capacity was measured according to a reported ORAC assay.^20^ The ORAC assay was carried out on a Synergy HT microplate fluorescence reader. The potassium phosphate buffer (75 mM, pH 7.4) was prepared with KH_2_PO_4_ and K_2_HPO_4_. 20 μL of the serial-diluted sample or Trolox was mixed with 160 μL of fluorescein solution (8.16 × 10^−5^ mM in buffer) in a 96-well plate. 20 μL of AAPH solution (153 mM in buffer) was automatically added into the plate to quench the fluorescence. The fluorescence was measured every minute for 2 h at 37 °C. The excitation wavelengths vary from 465 to 505 nm, and emission wavelengths vary from 505 to 555 nm.

### 2.7 Statistical analysis

Statistical analyses were carried out with an independent sample *t*-test and one-way analysis of variance (ANOVA) using IBM SPSS Statistics V.22.0 (IBM Corporation, Armonk, NY). All analyses were performed in triplicate and results were expressed as mean ± standard deviation. The significance level was set at 0.05.

## 3. Results and discussion

### 3.1 Physicochemical analysis

The physicochemical parameters of unripe and ripe fruit are listed in the Table 1. Among the nutritive compositions of fruit, the moisture contents (79.5% and 80.7%) accounted for the largest portion of the unripe and ripe fruit, which were no significant difference (*p* > 0.05 by *t*-test) as fruit ripen. Among the other compositions, the total dietary fibre was responsible for the majority of dried fruit, which was 59% of unripe fruit and 58% of ripe fruit. The total dietary fibre of Malay cherry fruit was higher than most of the fruit. For example, it was around 5 times higher than that of ripe apples.^21^ Dietary fibre is a necessary nutrient in a healthy diet, because it can ease constipation, reduce harmful substance levels (*e.g.* cholesterol and heavy metals), and prevent large intestine cancer, obesity, diabetes, and coronary heart diseases.^22^ Although there was no significant difference in the total dietary fibre, it is worth mentioning that the soluble dietary fibre significantly (*p* < 0.05) increased from 13.5% to 16.2% as fruit ripen. However, the insoluble dietary fibre significantly decreased from 45.7% to 41.4% as fruit ripen, which may due to the partial degradation of cellulose in the plant cell wall. The cellulose, a main insoluble fibre in fruit, could be digested by cellulase into monosaccharides, thereby softening the fruit during ripening.^23^ There was a rise in the TSS of fruit from 12.76% to 18.71% of ripen fruit. As a result, the total sugar significantly increased as fruit ripen. The total protein and total fat slightly increased during fruit ripening. Total ash contents did not experience a significant change as fruit ripen. The total carbohydrate can be estimated as the summation of total sugar, total dietary fibre, and total starch (starch was not detectable in the fruit).

**Table:** Physicochemical parameters for unripe and ripe Malay cherry fruit^a^

Physicochemical parameters	Unripe fruit	Ripe fruit
**Nutritive compositions (%)**		
Moisture	79.5 ± 0.7^a^	80.7 ± 0.3^a^
Total ash	4.6 ± 0.1^a^	4.1 ± 0.3^a^
Total protein	3.30 ± 0.05^b^	3.56 ± 0.05^a^
Total fat	2.25 ± 0.08^b^	2.8 ± 0.1^a^
Total sugar	5.91 ± 0.05^b^	7.3 ± 0.2^a^
Insoluble dietary fibre	45.7 ± 0.5^a^	41.4 ± 0.5^b^
Soluble dietary fibre	13.5 ± 0.6^b^	16.2 ± 0.6^a^
Total dietary fibre	59 ± 1^a^	58 ± 1^a^
Total soluble solids (%)	12.76 ± 0.01^b^	18.71 ± 0.01^a^
Reducing sugar (mg maltose equivalent per g dry weight of fruit)	166 ± 2^b^	184 ± 1^a^

**Sugar (mg g** ^**−1**^ **dry weight of fruit)**		
Fructose	22.7 ± 0.2^b^	29.6 ± 0.7^a^
Glucose	17.8 ± 0.7^b^	23.4 ± 0.3^a^
Sucrose	18.6 ± 0.3^a^	20 ± 1^a^

**Organic acid (mg g** ^**−1**^ **dry weight of fruit)**		
Malic acid	23.1 ± 0.8^a^	18.3 ± 0.8^b^
Succinic acid	11.5 ± 0.6^a^	5.7 ± 0.6^b^
Citric acid	9.0 ± 0.3^a^	2.2 ± 0.2^b^
pH	5.58 ± 0.01^b^	5.71 ± 0.04^a^

**Colour**		
L*	60 ± 1^a^	24.4 ± 0.5^b^
a*	−7.8 ± 0.2^b^	10 ± 2^a^
b*	30 ± 1^a^	2.4 ± 0.3^b^

**Dimension (cm)**		
Length	2.6 ± 0.3^a^	2.8 ± 0.2^a^
Diameter	2.2 ± 0.3^b^	3.0 ± 0.3^a^
Weight (g)	7.5 ± 0.6^b^	16 ± 3^a^

a Means of parameters within rows followed by different letters are significantly different (*p* < 0.05) according to the independent sample *t*-test. Values are means ± standard deviations (*n* = 3). Values are expressed on dry weight of fruit, except for the values of moisture on fresh fruit weight.

The main sugars found in the Malay cherry fruit were fructose, glucose, and sucrose. The fructose level was always higher than glucose and sucrose in response to various stresses.^24^ As fruit ripen, the fructose and glucose significantly increased from 22.7 and 17.8 to 29.6 and 23.4 mg g^−1^ dry weight of fruit, while the sucrose content slightly increased from 18.6 to 20 mg g^−1^ dry weight of fruit. Therefore, the total sugar and reducing sugar (fructose and glucose) of fruit experienced significant increases as fruit ripen. Similarly, an increase in sugars (fructose, glucose, total sugar, and reducing sugar) as sweet cherries ripen.^25^ The principle organic acid was malic acid in Malay cherry fruit, while the second and the third major acids were succinic and citric acids. This finding was agreement with the organic acids in sweet cherries.^26^ The three organic acids were high in unripe fruit and decrease in ripe fruit. Malay cherry fruit reflected a significant increase in pH as fruit ripen. In conclusion, the major compositions changes in ripening fruit are a significant increase in reducing sugar and a significant fall in organic acids.

The green colour of unripe fruit was as follows: L* = 60, a* = −7.8, b* = 30. The dark reddish-purple colour of ripe fruit (L* = 24.4, a* = 10, b* = 2.4) was from anthocyanins, which are pigments in the plants. Moreover, during ripening, the fruit dimensions increased from 2.6 to 2.8 cm in length and from 2.2 to 3.0 cm in diameter; the fruit weight increased from 7.5 to 16 g.

### 3.2 Structural characterisation of anthocyanins

**Fig. 1:**
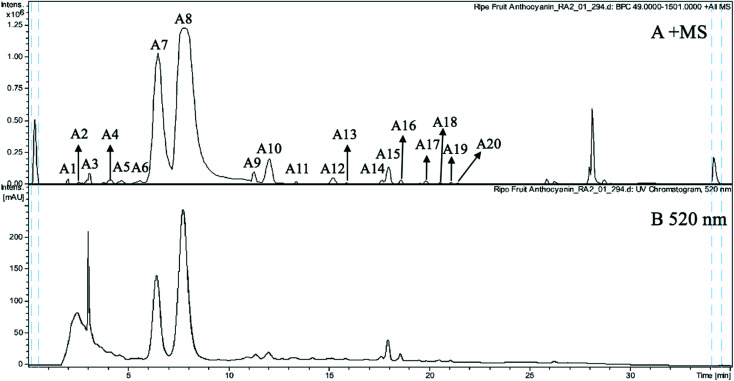
(A) MS spectrogram (in positive ion mode) and (B) HPLC chromatogram (UV 520 nm) of anthocyanins from ripe Malay cherry fruit.

The anthocyanin pigments found only in the ripe fruit were characterised by HPLC-HR-TOF/MS^2^ spectroscopy (Fig. 1A and B) and the corresponding MS profile data are summarised in Table 2. The chemical structures of anthocyanins are shown in the Fig. 3.

HPLC-PDA and HPLC-HR-TOF/MS^2^ of phenolic compounds of Malay cherry fruit^a^

**Table:** 

Anthocyanins	RT (min)	Tentative assignment	Chemical formula	MW	MS (*m*/*z*) [M]^+^	MS^2^ (*m*/*z*)	Error (ppm)	Exact mass (*m*/*z*)	HPLC-PDA *λ*_max_ (nm)	Ripe fruit	Unripe fruit
A1	2.0	Unknown	C_5_HN_4_O_12_^+^	308	308.9582	297.8974	1.1	308.9585	525, 278	+	−
A2	2.5	Cyanidin-3-*O*-sophoroside	C_27_H_31_O_16_^+^	611	611.1577	287.0565	4.9	611.1607	526, 278	+	−
A3	3.0	Cyanidin-3-*O*-glucosylrutinoside	C_33_H_41_O_20_^+^	757	757.2188	611.4959, 287.0565	−0.4	757.2186	516, 279	+	−
A4	4.0	Cyanidin-3,5-*O*-diglucoside	C_27_H_31_O_16_^+^	611	611.1607	287.0555, 449.1098	−0.1	611.1607	508, 278	+	−
A5	4.6	Cyanidin-3-*O*-rutinoside-5-*O*-glucoside	C_33_H_41_O_20_^+^	757	757.2179	595.1640, 449.1074, 287.0563	0.9	757.2186	516, 278	+	−
A6	5.5	Delphinidin-3-*O*-neohesperidoside	C_27_H_31_O_16_^+^	611	611.1597	303.0508	1.6	611.1607	517, 278	+	−
A7	6.5	Cyanidin-3-*O*-glucoside	C_21_H_21_O_11_^+^	449	449.1087	287.0570	−2.0	449.1078	515, 328, 280	+	−
A8	7.8	Cyanidin-3-*O*-rutinoside	C_27_H_31_O_15_^+^	595	595.1657	287.0568, 449.1083	0.0	595.1657	514, 329, 280	+	−
A9	11.2	Peonidin-3-*O*-glucoside	C_22_H_23_O_11_^+^	463	463.1232	301.0722	0.7	463.1235	519, 279	+	−
A10	12.0	Cyanidin-3-*O*-pentoside	C_20_H_19_O_10_^+^	419	419.0965	287.0559	1.8	419.0973	517, 279	+	−
A11	13.3	Cyanidin-3-*O*-(2′′′-acetylrutinoside)	C_29_H_33_O_16_^+^	637	637.1783	287.0559	−3.0	637.1763	525, 276	+	−
A12	15.2	Cyanidin-3-*O*-(6′′-acetylglucoside)	C_23_H_23_O_12_^+^	491	491.1180	287.0550	0.7	491.1184	525, 275	+	−
A13	15.8	Delphinidin-3,5-*O*-diglucoside	C_27_H_31_O_17_^+^	627	627.1558	303.0504, 465.0924	−0.4	627.1556	519, 279	+	−
A14	17.6	Delphinidin-3-*O*-(6′′-coumaroylglucoside)	C_27_H_31_O_16_^+^	611	611.1606	303.0501	0.1	611.1607	525, 281	+	−
A15	18.0	Delphinidin-3-*O*-rutinoside	C_27_H_31_O_16_^+^	611	611.1596	303.0501	1.7	611.1607	525, 281	+	−
A16	18.6	Cyanidin-3-*O*-glucoside-5-*O*-pentoside	C_26_H_29_O_15_^+^	581	581.1375	449.1254, 419.1015, 287.0796	> 5.0	581.1501	525, 281	+	−
A17	19.8	Unknown	C_18_H_30_NO_10_^+^	420	420.1866	240.1006	−0.4	420.1864	—	+	−
A18	20.5	Cyanidin-3-*O*-glucoside-7-*O*-rhamnoside	C_27_H_31_O_15_^+^	595	595.1677	287.0552	−3.4	595.1657	—	+	−
A19	21.0	Petunidin-3-*O*-rutinoside	C_28_H_33_O_16_^+^	625	625.1747	317.0668	2.5	625.1763	—	+	−
A20	21.4	Unknown	C_21_H_31_O_9_^+^	427	427.1971	240.1016	−2.0	427.1963	—	+	−

a +, detected; −, not detected.

**Table:** 

Non-anthocyanin phenolics	RT (min)	Tentative assignment	Chemical formula	MW	MS (*m*/*z*) [M − H]^−^	MS^2^ (*m*/*z*)	Error (ppm)	Exact mass (*m*/*z*)	HPLC-PDA *λ*_max_ (nm)	Ripe fruit	Unripe fruit
P1	1.7	Unknown	C_16_H_32_O_2_	256	255.2316	—	5.4	255.2330	—	+	+
P2	2.5	Caffeic acid-4-*O*-glucoside	C_15_H_18_O_9_	342	341.1064	—	>10	341.0878	244	+	+
P3	3.4	Methyl 4-*O*-galactopyranosyl-2,3-di-*O*-methyl-galactopyranoside	C_15_H_28_O_11_	384	383.1547	—	3.2	383.1559	232	+	+
P4	3.6	Mangiferdiol	C_21_H_24_O_12_	468	467.1218	287.0584	−4.8	467.1195	233	+	+
P5	4.5	Unknown	C_30_H_30_O_3_	438	437.2123	218.1074	−0.1	437.2122	233	+	+
P6	5.2	3-Isopentadienyl-3′,4,5′-trihydroxystilbene	C_19_H_18_O_3_	294	293.1205	113.3011	−7.4	293.1183	273	+	−
P7	5.9	(Epi)gallocatechin-(epi)catechin	C_30_H_26_O_13_	594	593.1327	407.0740, 289.0685	−4.5	593.1301	286	+	−
P8	6.5	Unknown	C_17_H_32_O_12_	428	427.1822	367.1615	−0.2	427.1821	278	+	+
P9	7.0	Eriodictyol-7-*O*-rutinoside	C_27_H_32_O_15_	596	595.1675	287.0575	−1.0	595.1668	281	+	−
P10	7.6	Eriodictyol-*O*-hexoside I	C_21_H_22_O_11_	450	449.1088	287.6681	0.4	449.1089	283	+	−
P11	8.8	Taxifolin-3-*O*-hexoside	C_21_H_22_O_12_	466	465.1047	303.0512	−1.8	465.1038	286	+	+
P12	9.4	Benzyl alcohol-hexoside-pentoside I	C_18_H_26_O_10_	402	401.1462	269.0992	−2.2	401.1453	291	+	−
P13	10.0	Verbasoside	C_20_H_30_O_12_	462	461.1680	269.0987	−3.3	461.1664	285	+	−
P14	10.5	Benzyl alcohol-hexoside-pentoside II	C_18_H_26_O_10_	402	401.1448	269.1046	1.3	401.1453	273	+	+
P15	10.7	Primulaverin	C_20_H_28_O_13_	476	475.1439	—	3.9	475.1457	284	−	+
P16	11.6	Procyanidin dimer	C_30_H_26_O_12_	578	577.1356	407.0767, 289.0683	−0.7	577.1351	280	+	+
P17	14.6	(Epi)catechin	C_15_H_14_O_6_	290	289.0711	221.0824	2.2	289.0718	279	+	+
P18	15.2	Eriodictyol-*O*-hexoside II	C_21_H_22_O_11_	450	449.1087	287.0550	0.6	449.1089	287	+	+
P19	15.5	Eriodictyol-*O*-hexoside III	C_21_H_22_O_11_	450	449.1090	287.0582	−0.2	449.1089	287	−	+
P20	16.3	Neobavaisoflavone	C_20_H_18_O_4_	322	321.1151	—	−5.7	321.1132	281	+	−
P21	16.6	Vanillic acid-4-*O*-glucoside	C_14_H_18_O_9_	330	329.0863	209.0509	4.6	329.0878	281	+	+
P22	18.2	Primeveroside	C_19_H_28_O_10_	416	415.1627	—	−4.1	415.1610	285	+	−
P23	18.6	(Epi)catechin-(epi)gallocatechin	C_30_H_26_O_13_	594	593.1296	407.0779, 289.0730	0.9	593.1301	286	+	+
P24	19.6	Jasminoside R	C_22_H_34_O_12_	490	489.1987	—	−2.0	489.1978	283	+	+
P25	20.6	Myricetin-3-*O*-rutinoside	C_27_H_30_O_17_	626	625.1412	317.0226	−0.2	625.1410	272	+	+
P26	21.3	Ferulic acid-4-*O*-glucoside	C_16_H_20_O_9_	356	355.1024	193.3516	2.9	355.1035	283	+	+
P27	22.3	(2Z)-6-[5-(β-d-Glucopyranosyloxy)-4-hydroxy-2-methylphenyl]-2-methyl-2-heptenoic acid	C_21_H_30_O_9_	426	425.1813	219.1380	0.9	425.1817	275	+	+
P28	22.7	Quercetin-3-*O*-rutinoside	C_27_H_30_O_16_	610	609.1457	301.0333	0.6	609.1461	257, 354	+	+
P29	23.4	Quercetin-3-*O*-glucoside	C_21_H_20_O_12_	464	463.0889	301.0321	−1.6	463.0882	257, 291, 354	+	+
P30	24.2	Kaempferol-3-*O*-rutinoside	C_27_H_30_O_15_	594	593.1514	285.0375	−0.3	593.1512	267, 286, 344	+	+
P31	24.4	Isorhamnetin-3-*O*-rutinoside	C_28_H_32_O_16_	624	623.1618	315.0423	0.0	623.1618	267, 354	−	+
P32	25.0	Luteolin-7-*O*-hexoside	C_21_H_20_O_11_	448	447.0921	285.0367	2.7	447.0933	293	+	+
P33	26.1	Astringin	C_20_H_22_O_9_	406	405.1197	—	−1.4	405.1191	283, 373	+	+
P34	26.6	Quercetin-3-*O*-rhamnoside	C_21_H_20_O_11_	448	447.0921	301.0332	2.8	447.0933	280	+	+
P35	27.2	Quercetin	C_15_H_10_O_7_	302	301.0361	—	−2.4	301.0354	281	+	+
P36	28.3	Quercetin-4′-*O*-galactoside	C_20_H_18_O_12_	450	449.0759	363.0729	−7.5	449.0725	271, 375	+	−
P37	30.7	Unknown	C_12_H_24_O_4_	232	231.1604	—	−0.9	231.1602	292	+	−
P38	31.2	Unknown	C_18_H_32_O_5_	328	327.2168	—	2.9	327.2177	273	+	+
P39	32.5	Pinellic acid	C_18_H_34_O_5_	330	329.2325	209.1199	2.6	329.2333	283	+	+

Peak A2 produced a cationic *m*/*z* 611 [M]^+^ and a fragment *m*/*z* 287 [M − 324]^+^ (due to the loss of a sophoroside moiety), which was tentatively identified as cyanidin-3-*O*-sophoroside. The *m*/*z* 287 is typical for the cyanidin moiety. Peak A4 also gave a cationic *m*/*z* 611 [M]^+^ but the fragments were *m*/*z* 287 [M − 324]^+^ and *m*/*z* 449 [M − 162]^+^ (due to the loss of a glucoside moiety), and thus it was tentatively identified as cyanidin-3,5-*O*-diglucoside.^27^ Peaks A6, A14, and A15 shared the same cationic *m*/*z* 611 [M]^+^ and the same fragment *m*/*z* 303 [M − 308]^+^. The *m*/*z* 303 is typical for the delphinidin moiety. The 308 Da is related to different glycoside moieties. Therefore, peaks A6, A14, and A15 were tentatively identified as delphinidin-3-*O*-neohesperidoside, delphinidin-3-*O*-(6′′-coumaroylglucoside), and delphinidin-3-*O*-rutinoside, respectively.^28^

Peaks A3 and A5 gave the same cationic *m*/*z* 757 [M]^+^. The major fragments of peak A3 were *m*/*z* 611 [M − 146]^+^ (due to the loss of a rhamnoside moiety) and *m*/*z* 287 [M − 146−162 − 162]^+^. The peak A3 was thus tentatively identified to the fragment profile of cyanidin-3-*O*-glucosylrutinoside. The major fragments of peak A5 were *m*/*z* 595 [M − 162]^+^, *m*/*z* 449 [M − 162 − 146]^+^, and *m*/*z* 287 [M − 162 − 146 − 162]^+^. Therefore, the peak A5 was tentatively identified as cyanidin-3-*O*-rutinoside-5-*O*-glucoside. The difference between the two peaks was the loss of glycoside moieties in different sequences.

Peaks A8 and A18 had cationic *m*/*z* 595 [M]^+^. Peak A8 fragmented into *m*/*z* 449 [M − 146]^+^ and *m*/*z* 287 [M − 146 − 162]^+^, which was consistent with and tentatively identified as cyanidin-3-*O*-rutinoside.^27^ The peak A18 has fragmentation pattern cyanidin-3-*O*-rutinoside with one fragment at *m*/*z* 287, and thus it was tentatively assigned as cyanidin-3-*O*-glucoside-7-*O*-rhamnoside.

Peaks A7 (*m*/*z* 449), A10 (*m*/*z* 419), A11 (*m*/*z* 637), and A12 (*m*/*z* 491) yielded the same fragment *m*/*z* 287, which equates with cyanidin moiety and derives after the removal of glucoside moiety (162 Da), pentoside moiety (132 Da), acetylrutinoside moiety (350 Da), and acetylglucoside moiety (204 Da) from the [M]^+^. Peaks A7, A10, A11, and A12 were thus tentatively assigned as cyanidin-3-*O*-glucoside, cyanidin-3-*O*-pentoside, cyanidin-3-*O*-(2′′′-acetylrutinoside), and cyanidin-3-*O*-(6′′-acetylglucoside), respectively.

Peak A9 yielded *m*/*z* 463 [M]^+^ which fragmented into *m*/*z* 301 and a glucosyl moiety. It is consistent with peonidin. Therefore, peak A9 was tentatively assigned as peonidin-3-*O*-glucoside.

Peak A13 shown a *m*/*z* 627 [M]^+^ and was tentatively assigned as delphinidin-3,5-*O*-diglucoside. The fragment *m*/*z* 303 corresponds to the delphinidin aglycone and the lost neutral fragment of 324 Da corresponds to two glucoside (162 Da) moieties.

Peak A19 was tentatively assigned as petunidin-3-*O*-rutinoside, because it produced *m*/*z* 625 [M]^+^ and yielded a fragment at *m*/*z* 317 [M − 308]^+^. The 308 Da corresponds to the rutinoside moiety. Peak A16 was tentatively assigned as cyanidin-3-*O*-glucoside-5-*O*-pentoside because it had a molecular cation at *m*/*z* 581, which fragmented into *m*/*z* 449 [M − 132]^+^ and 419 [M − 162]^+^.

The indicator of fruit ripening is reddish purple colour development, which results from the accumulation of anthocyanins. In sweet cherries, cyanidin-3-*O*-glucoside and cyanidin-3-*O*-rutinoside were found as major anthocyanins and exerted high antioxidant capacities, while tart cherries were rich in cyanidin-3-*O*-glucosylrutinoside and cyanidin-3-*O*-rutinoside.^29^ However, more varieties of anthocyanins were found in Malay cherry fruit. The subtle structural variations of anthocyanins may dramatically vary their bioactivity. Therefore, it is of great interest to study the health promoting functions of the anthocyanins from Malay cherry fruit.

### 3.3 Structural characterisation of non-anthocyanin phenolics

**Fig. 2:**
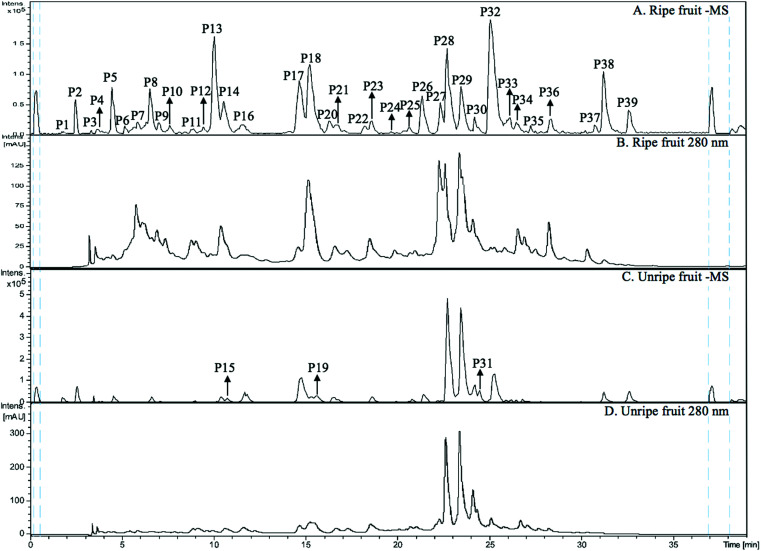
(A) HPLC-HR-TOF/MS^2^ trace (in negative ion mode) and (B) HPLC chromatogram (UV 280 nm) of non-anthocyanin phenolics from ripe Malay cherry fruit; (C) HPLC-HR-TOF/MS^2^ trace (in negative ion mode) and (D) HPLC chromatogram (UV 280 nm) of non-anthocyanin phenolics from unripe Malay cherry fruit.

**Fig. 3:**
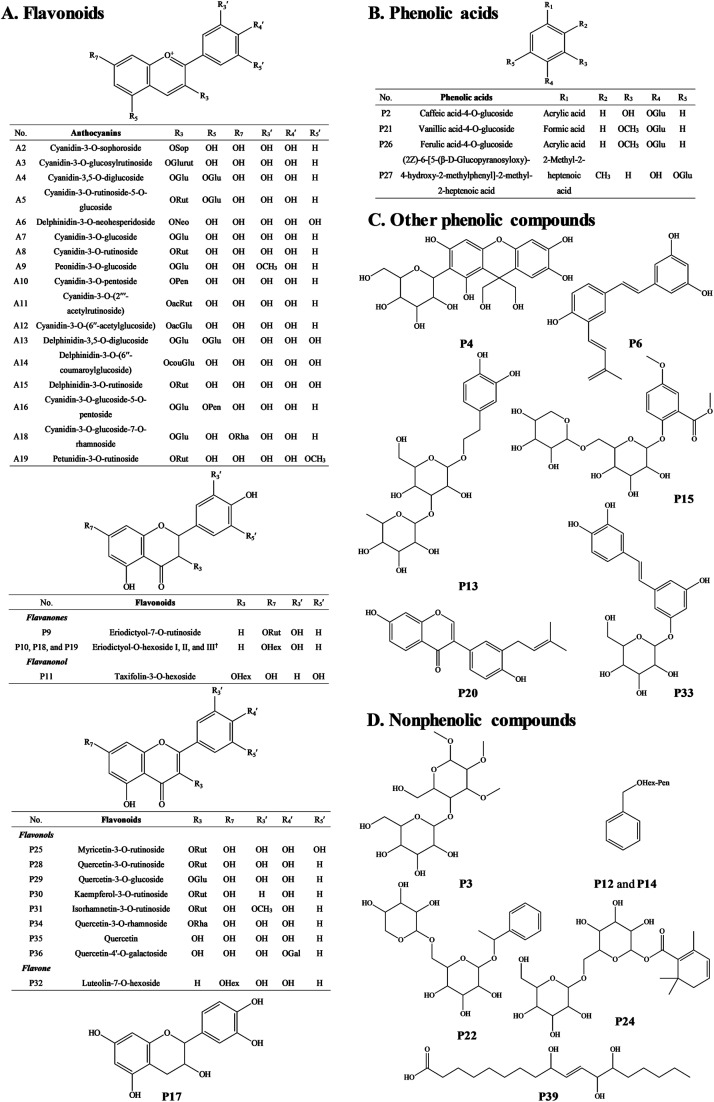
The chemical structures of (A–C) phenolic and (D) nonphenolic compounds characterised in unripe and ripe Malay cherry fruit. Abbreviations: Sop, sophoroside; Glurut, glucosylrutinoside; Glu, glucoside; Rut, rutinoside; Neo, neohesperidoside; Pen, pentoside; Rha, rhamnoside; ac, acetyl; cou, coumaroyl; Hex, hexoside; Gal, galactoside. ^†^Eriodictyol-7-*O*-hexoside was taken as a structural example for the eriodictyol-*O*-hexoside I, II, and III.

The MS spectra and HPLC chromatogram of non-anthocyanin fraction from unripe and ripe Malay cherry fruit are shown in Fig. 2, and the corresponding MS profile data are summarised in Table 2. The chemical structures of identified compounds in Malay cherry fruit are shown in Fig. 3. Among the identified compounds, majority of them are flavonoids along with some nonphenolic compounds and unknown structures.

#### 3.3.1 Flavanones

Peak P9 shown a molecular anion at *m*/*z* 595 [M − H]^−^, while peaks P10, P18, and P19 produced the *m*/*z* 449 [M − H]^−^. Their main fragment was *m*/*z* 287, which corresponded to eriodictyol after the loss of a rutinoside moiety (308 Da) from P9 or the loss of a hexoside moiety (162 Da) from P10, P18, and P19. Therefore, peak P9 was tentatively identified as eriodictyol-7-*O*-rutinoside,^30^ while peaks P10, P18, and P19 were tentatively identified as eriodictyol-*O*-hexoside isomers.^31^

#### 3.3.2 Flavanonols

Peak P11 showed a parent ion at *m*/*z* 465 [M − H]^−^. The parent ion yielded a fragment at *m*/*z* 303 [M − H − 162]^−^, which corresponded to a taxifolin moiety. The 162 Da is typical for a hexoside moiety. The peak P11 was tentatively identified as taxifolin-3-*O*-hexoside.^32^

#### 3.3.3 Flavonols

Peak P25 gave a parent ion at *m*/*z* 625 [M − H]^−^ and a fragment ion at *m*/*z* 317 [M − H − 308]^−^ (loss of a rutinoside moiety). The *m*/*z* 317 was related to the myricetin moiety. The peak P25 was tentatively identified as myricetin-3-*O*-rutinoside. Peak P28 was tentatively characterised as quercetin-3-*O*-rutinoside exhibiting an [M − H]^−^ ion at *m*/*z* 609 and a fragment ion at *m*/*z* 301 [M − H − 308]^−^. The 301 Da (Peak P35) was related to a quercetin moiety after the loss of a rutinoside moiety (308 Da). Peak P29 exhibited an [M − H]^−^ ion at *m*/*z* 463 and a fragment ion at *m*/*z* 301 [M − H − 162]^−^. Thus, it was tentatively characterised as quercetin-3-*O*-glucoside.^31^ Peak P34 was quercetin-3-*O*-rhamnoside exhibiting an [M − H]^−^ ion at *m*/*z* 447 and a fragment ion at *m*/*z* 301 [M − H − 146]^−^ (loss of a rhamnoside moiety).^33^ Peak P36 was tentatively characterised as quercetin-4′-*O*-galactoside. The Peaks P30 ([M − H]^−^, *m*/*z* 593) and P31 ([M − H]^−^, *m*/*z* 623) were tentatively characterised as kaempferol-3-*O*-rutinoside and isorhamnetin-3-*O*-rutinoside exhibiting their fragment ion at *m*/*z* 285 (kaempferol moiety) and *m*/*z* 315 (isorhamnetin moiety), respectively.

Peak P32 was tentatively identified as luteolin-7-*O*-hexoside (Flavone) while peak P17 with a characteristic ion [M − H]^−^ at *m*/*z* 289 was corresponded to catechin or epicatechin (Flavanol). Its fragment *m*/*z* 221 [M − H − 68]^−^ was due to a loss of C_3_O_2_.

#### 3.3.4 Proanthocyanidins

Peak P16 ([M − H]^−^, *m*/*z* 577) yielded fragment ions at *m*/*z* 407 ([M − H − 170]^−^) from retro-Diels–Alder fission of the heterocyclic ring and loss of a water molecule, and at *m*/*z* 289 ([M − H − 289]^−^) from cleavage of the interflavanyl bond. Thus, peak P16 contained two (epi)catechins, which was a procyanidin dimer. Peaks P7 and P23 gave the same anionic *m*/*z* 593 [M − H]^−^, and yielded two fragments *m*/*z* 407 [M − H − 186]^−^ (loss of C_8_H_8_O_4_ and H_2_O) and *m*/*z* 289 [M − H − 304]^−^. The ion peak at *m*/*z* 593 suggested that P7 and P23 were proanthocyanidins consisting of one (epi)catechin and one (epi)gallocatechin.^34^

#### 3.3.5 Phenolic acids

Peak P2, which produced an anionic *m*/*z* 341 [M − H]− and its chemical formula was calculated as C_15_H_18_O_9_. Therefore, the peak P2 was tentatively identified as caffeic acid-4-*O*-glucoside. Peaks P21 and P26 also contained the glucoside moiety, and thus they were tentatively identified as vanillic acid-4-*O*-glucoside and ferulic acid-4-*O*-glucoside. Peak P27 was tentatively characterised as (2*Z*)-6-[5-(β-d-glucopyranosyloxy)-4-hydroxy-2-methylphenyl]-2-methyl-2-heptenoic acid.

#### 3.3.6 Other phenolic compounds

Peak P4 gave an anionic *m*/*z* 467 [M − H]^−^ (calculated for C_21_H_23_O_12_, 467.1195) and a fragment *m*/*z* 287 [M − H − 180]^−^ (due to the loss of a glucoside moiety and a water molecule), which was tentatively identified as mangiferdiol. The mangiferdiol is a mangiferin analogue. The xanthone glucoside mangiferin belongs to polyphenols.^35^ Peak P6 produced an anionic *m*/*z* 293 [M − H]^−^ (calculated for C_19_H_17_O_3_, 293.1183) and a fragment *m*/*z* 113 [M − H − 180]^−^, which was tentatively identified as 3-isopentadienyl-3′,4,5′-trihydroxystilbene.^36^ Peaks P13, P15, P20, and P33 were tentatively characterised as verbasoside, primulaverin, neobavaisoflavone, and astringin, respectively based on the HRMS peak for molecular ions and their fragmentation patterns.

#### 3.3.7 Nonphenolic compounds

Peak P3 produced an anionic *m*/*z* 383 [M − H]^−^ and its chemical formula was calculated as C_15_H_28_O_11_. The peak P3 was tentatively identified as methyl 4-*O*-galactopyranosyl-2,3-di-*O*-methyl-galactopyranoside. Peaks P12 and P14 produced a parent ion at *m*/*z* 401 [M − H]^−^. The parent ion yielded a fragment at *m*/*z* 269 [M − H − 132]^−^, which was due to the loss of a pentoside moiety (132 Da). They were tentatively identified as benzyl alcohol-hexoside-pentoside isomers.^37^ Peak P22 was tentatively characterised as primeveroside. Peak P24 was tentatively characterised as jasminoside R. Peak P39 was tentatively identified as oxylipin, pinellic acid.^38^

### 3.4 Quantification of total phenolic, anthocyanin, and proanthocyanidin contents

Table 3 shows the TPC, TAC, and TPAC of unripe and ripe fruit. The TPC of ripe fruit (36.5 ± 0.3 mg gallic acid equivalent per g of dry weight of fruit) showed significantly (*p* < 0.05 by *t*-test) lower than that of unripe fruit (92 ± 3 mg gallic acid equivalent per g of dry weight of fruit). A significant fall also can be found in the TPAC as fruit ripen. The polyphenols, especially proanthocyanidins, are responsible for the bitter and astringent tastes.^39^ As fruit ripen, the decrease in bitterness and astringency in fruit can explain the decrease in TPC and TPAC.^40^ In addition, the higher TPC and TPAC in unripe fruit can be attributed to the higher rates of metabolite biosynthesis and protection against invasive actions of external organisms (*i.e.* diseases and insect pests) and adverse environmental conditions (*i.e.* UV light) at the earlier age of fruit.^41^ Similarly, the decreases in TPC and TPAC as fruit ripen were reported in dates, apples,^42^ and grapes.^43^ However, the TPC of Malay cherry fruit was higher than most of other fruit, such as tart cherries, sweet cherries, and blueberries, and thus Malay cherry fruit can be served as rich sources of phenolic compounds in a healthy diet.^44^ Anthocyanins are synthesised as fruit ripen, resulting in the development of a reddish purple colour. Consistent with the visual colour change, TAC increased markedly from unripe (not detected) to ripe stages (1.32 ± 0.02 mg cyanidin-3-*O*-glucoside equivalent per g of dry weight of fruit).

**Table:** The antioxidant capacity, total phenolic, anthocyanin, and proanthocyanidin contents of Malay cherry fruit^a^

Maturity	SPE fraction	ORAC^b^ (μmol Trolox equivalent per g dry weight of fraction)	TPC^c^ (mg gallic acid equivalent per g of dry weight of fruit)	TAC (mg cyanidin-3-*O*-glucoside equivalent per g of dry weight of fruit)	TPAC^c^ (mg procyanidin A2 equivalent per g of dry weight of fruit)
Unripe fruit	Crude extracts	487.17 ± 28.06^d^	92 ± 3^a^	—	18 ± 1^a^
	Water fraction	44 ± 5^d^			
	Non-anthocyanin phenolics	4584 ± 481^a^			
	Anthocyanins	168 ± 17^d^			
Ripe fruit	Crude extracts	204 ± 4^d^	36.5 ± 0.3^b^	1.32 ± 0.02	6.4 ± 0.1^b^
	Water fraction	30 ± 3^d^			
	Non-anthocyanin phenolics	2254 ± 57^b^			
	Anthocyanins	1031 ± 81^c^			

a –, not detected; values are means ± standard deviations (*n* = 3).

b Means values with different letters in the column are significantly different by analysis of variance Tukey test in one-way independent groups design (*p* < 0.05).

c Means values with different letters in the same column are significantly different by independent sample *t*-test (*p* < 0.05).

### 3.5 Antioxidant capacity

The results for antioxidant capacities of unripe and ripe fruit are shown in Table 3. The crude extracts of unripe and ripe fruit were purified using SPE and obtained three fractions, which were water fraction, non-anthocyanin phenolics, and anthocyanins in sequence. The non-anthocyanin phenolics of unripe fruit had the significantly (*p* < 0.05 by ANOVA) highest antioxidant capacities at ORAC value of 4584 μmol Trolox equivalent per g dry weight of fraction, followed by the non-anthocyanin phenolics of ripe fruit (2254 μmol Trolox equivalent per g of dry weight of fruit). The anthocyanins of ripe fruit had medium antioxidant capacity (1031 μmol Trolox equivalent per g of dry weight of fruit). The crude extracts, water fraction, and anthocyanins of unripe fruit had low antioxidant capacities. Therefore, it shows that the non-anthocyanin phenolics were the main antioxidants in Malay cherry fruit, and the non-anthocyanin phenolics of unripe fruit showed significantly higher antioxidant capacity than ripe fruit. The above findings implied that the TPC of Malay cherry fruit generally decreased as fruit ripen. In many cases, antioxidant capacity, as well as TPC of berries, such as blueberry^45^ and strawberry,^46^ tended to decrease as fruit ripen.

## 4. Conclusions

From our results, nutritive compositions (sugars, proteins, and fats) of Malay cherry fruit increased, whilst organic acids of fruit decreased upon ripening. The antioxidant capacities of fruit decreased, as the polyphenols of fruit decreased upon ripening. It is apparent that Malay cherry fruit has high fibre contents and is rich in polyphenolic antioxidants of diverse structures motifs that are known to have health promotion activity. Therefore, Malay cherry fruit are a good addition to the family of cherries and berries that have been considered as superfoods for human health. The ripening of Malay cherry fruit would be of particular interest to the food industry, as different agricultural practices could obviously affect the levels of beneficial effects that could be obtained from consuming their polyphenolic extracts of different ripening stages. It warrants further study on cellular and animal models, to establish scientific evidence of the bioactivity of the polyphenolic compounds extracted from Malay cherry fruit, particularly the unripe fruit.

## Conflicts of interest

There are no conflicts to declare.

## Supplementary Material
